# Thermal treadmills: how heat variability pushes salmon to their limits

**DOI:** 10.1093/conphys/coaf064

**Published:** 2025-08-14

**Authors:** Nathaniel Lambert

**Affiliations:** Department of Fish and Wildlife, Virginia Polytechnic Institute and State University, 310 W Campus Dr, Blacksburg, VA 24060, USA

Just how warm is too warm? Steelhead salmon (*Oncorhynchus mykiss)* do not all agree—it depends on where they live. A recent study performed by the University of California, Santa Barbara (Dressler *et al.*, 2025), reveals that a fish’s heat tolerance is shaped by the stream it calls home. However, every fish has a limit, no matter where they are from.

As climate change causes rivers and streams to heat up, fisheries managers are under pressure to find which populations can cope with the heat and which are slipping past their tipping point. Some populations may be used to warmer waters, but they could already be living at the edge of what they can survive.

To pinpoint these thresholds, Terra Dressler and her team studied four Oregon waterways that vary in temperature but are all at the same latitude. They chose two warmer inland streams (the Lower Deschutes and John Day rivers), and two cooler coastal streams (the North Umpqua and Siletz). While all the streams differ in temperature ranges throughout the year, the biggest difference is in how much they differ day to day. The John Day, for example, has maximum daily temperature fluctuations (13°C) over three times that of the Siletz (4°C). That kind of temperature whiplash is like running on a treadmill that keeps changing speed—some fish cannot help but fall.

The researchers used several measures of stress and functional thermal tolerance: CT_MAX_ (the temperature at which fish lose equilibrium) served as a proxy for a lethal limit. They also included Absolute Aerobic Scope (energy available for basic functions past maintenance) and Exercise Recovery (how quickly metabolism returns to resting after intense exertion). Think of it like how long it takes us to catch our breath after a run; it’s harder on a hot, sunny day.

To minimize stress, the team took all measurements creek-side (Fig. 1). They slowly warmed tanks until fish lost balance, a sign they’d reached their temperature limit, and measured their oxygen uptake to determine their energetic capacity.

**Figure 1:**
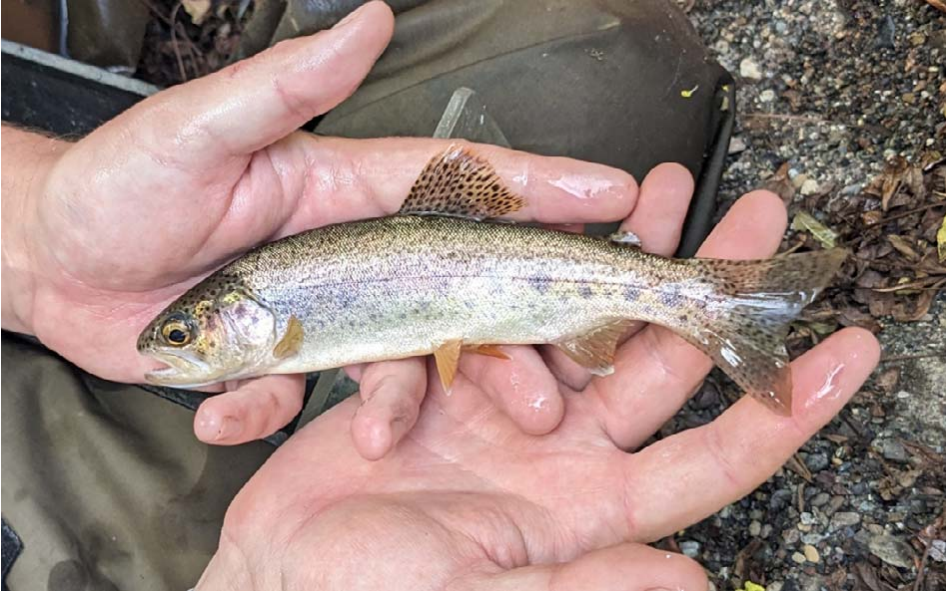
Steelhead salmon. Image credit: Terra Dressler

The results? Steelhead from warmer, inland streams already had higher resting metabolisms and could not handle much more heat stress. Many are currently living near their thermal breaking point where basic function like digestion, movement, and rest all become more difficult—and that safe range is only shrinking over time. In contrast, populations from cooler, coastal streams have more “breathing room.” They start with lower stress and have a greater buffer before conditions become dangerous.

So what does this mean for the fish? Not all salmon are equally prepared to handle rising temperatures. With limited conservation resources, we need to target the populations that need it most. The work done by Dressler and colleagues demonstrates that background climate shapes a fish’s physiological future. To protect this culturally iconic Pacific Northwest species, managers need to abandon the one-size-fits-all approach and apply conservation strategies with precision.

To protect Steelhead in our warming world, we must let physiology guide action—before the water gets too hot.

## Notes

**Editor:** Ashleigh Donaldson
